# Comparison of costs and discharge outcomes for patients hospitalized for ischemic or hemorrhagic stroke with or without atrial fibrillation in the United States

**DOI:** 10.1007/s11239-014-1144-8

**Published:** 2014-11-05

**Authors:** Xianying Pan, Teresa A. Simon, Melissa Hamilton, Andreas Kuznik

**Affiliations:** 1 Global Health Economics and Outcomes Research, Bristol-Myers Squibb Company, 5 Research Parkway, Wallingford, CT 06492 USA; 2 Global Pharmacovigilence and Epidemiology, Bristol-Myers Squibb Company, Hopewell, NJ 08534 USA; 3 Global Health Economics and Outcomes Research, Bristol-Myers Squibb Company, 100 Nassau Park Boulevard, 3.NN37, Princeton, NJ 08543 USA; 4 Department of Pricing and Market Access, Celgene Corporation, 33 Technology Drive, Warren, NJ 07059 USA

**Keywords:** Atrial fibrillation, Hemorrhagic stroke, Ischemic stroke, Patient outcomes

## Abstract

This retrospective analysis investigated the impact of baseline clinical characteristics, including atrial fibrillation (AF), on hospital discharge status (to home or continuing care), mortality, length of hospital stay, and treatment costs in patients hospitalized for stroke. The analysis included adult patients hospitalized with a primary diagnosis of ischemic or hemorrhagic stroke between January 2006 and June 2011 from the premier alliance database, a large nationally representative database of inpatient health records. Patients included in the analysis were categorized as with or without AF, based on the presence or absence of a secondary listed diagnosis of AF. Irrespective of stroke type (ischemic or hemorrhagic), AF was associated with an increased risk of mortality during the index hospitalization event, as well as a higher probability of discharge to a continuing care facility, longer duration of stay, and higher treatment costs. In patients hospitalized for a stroke event, AF appears to be an independent risk factor of in-hospital mortality, discharge to continuing care, length of hospital stay, and increased treatment costs.

## Introduction

Atrial fibrillation (AF) is the most common cardiac arrhythmia in clinical practice, with an estimated 70 % of cases classified as nonvalvular AF [1]. A large US prospective cohort study estimated the lifetime risk of AF in people 50 years of age to be 25.9 % for men and 23.2 % for women [2]. The prevalence of AF in the United States in 2012 was estimated at 5.8 million based on data from a large database of US health insurance claims [3], and estimates based on Medicare data from 2007 to 2008 put the 2010 prevalence of nonvalvular AF at 5.3 % of the US Medicare population [1]. This figure is expected to increase over time, and estimates of the prevalence of AF in the United States in 2050 range from 5.61 million to 15.9 million [4, 5].

In the Framingham Heart Study, the risk of stroke in individuals with AF was five-fold greater than in those without AF, and was substantially higher than risks for other cardiovascular conditions [6]. Ischemic strokes account for 10 % of all deaths occurring within the first 4 months after a diagnosis of AF, and 7 % of deaths occurring after this period [7, 8]. In addition, the prevalence of AF and the risk of stroke increase with advancing age [6, 9]. Risk of stroke attributed to AF rises from 1.5 % at 50–59 years of age to 23.5 % at 80–89 years [6].

Strokes in individuals with AF are generally more severe and are associated with greater stroke-related mortality [10, 11]. AF-related stroke also raises the risks of recurrence, functional impairment, and being subsequently bedridden [10–12]. Thus, survivors of AF-related stroke are more likely to have disability and require greater inpatient and long-term care than stroke survivors without AF, all of which impact medical costs. Both ischemic and hemorrhagic strokes increase ongoing medical costs in patients with AF relative to patients with AF who have not experienced stroke, and the cost of care may remain elevated for several years after the event [13]. Patients with AF and additional risk factors for stroke have higher cardiovascular-related inpatient costs [14] and higher rates of rehospitalization, with readmissions incurring greater costs than the initial admission for AF [15]. The total US healthcare cost of all strokes was estimated at $53.9 billion in 2010 [16]. In turn, the cost of AF-related strokes was estimated to be as high as $13 billion [17].

Limited information is available comparing patient outcomes following stroke in patients with and without AF. The aim of this study was to evaluate and quantify the impact of AF on the discharge status, length of hospital stay, and associated costs for patients hospitalized for either ischemic or hemorrhagic stroke using data from a large US healthcare database.

## Methods

### Data source and study population

Patients were identified from the premier alliance database (Premier, Inc., Charlotte, NC, USA), a large nationally representative database (Premier is an alliance of community-based hospitals with over 2,700 hospital members) accounting for 5 million discharges annually. The analysis included all adult patients hospitalized with a primary diagnosis of ischemic stroke (ICD-9: 433.xx, 434.xx) or hemorrhagic stroke (ICD-9: 430.xx, 431.xx, 432.xx) between January 2006 and June 2011 (patients with multiple hospitalizations for stroke were excluded). Patients included in the analysis were identified as with or without AF, based on the presence or absence of a secondary listed diagnosis of AF (ICD-9: 427.3x). Additional secondary diagnoses were included using the Charlson comorbidity index [18] as a guide. These diagnoses were evaluated and included as comorbid conditions.

## Study design

This was a retrospective analysis investigating clinical characteristics, hospital discharge status (to home or continuing care), mortality, length of hospital stay, and patient costs associated with stroke in patients with or without AF. Ischemic and hemorrhagic strokes were investigated separately in the analysis.

### Statistical analysis

Data on length of hospital stay were analyzed using a Poisson regression model; patient cost and discharge status data were analyzed using generalized linear models with gamma distribution and log link, and multinomial distribution, respectively. The absolute differences in length of hospital stay and patient cost between AF and non-AF patients were the differences of least square means. All analyses were conducted using SAS version 9.2 (SAS Inc., Cary, NC, USA) software.

Demographic variables included sex, race (White, Black, Hispanic, other), and age (18–39, 40–49, 50–59, 60–69, 70–79, and 80+ years). Clinical variables included hypertension and variables from the Charlson comorbidity index [18]. Clinical variables that may have been associated with the index stroke event rather than preexisting stroke risk factors, such as hemiplegia and cerebrovascular disease, were excluded. Additional variables included hospital size (number of beds, categorized as 0–99, 100–199, 200–299, 300–399, 400–499, 500–599, 600–699, 700–799, 800–899, and 900+), hospital type (urban or rural; teaching or nonteaching), and geographic region (Midwest, Northeast, South, and West).

Using a simplified version of the discharge/mortality code included in the premier alliance database, status was characterized as “home,” “continuing care,” or “died during hospitalization.” The home code included all discharges from hospital to home. Continuing care was defined as discharge to skilled nursing facility, other hospital or hospital department, hospice, or other medical department or facility. All expired codes (indicating patient deaths) were included under died during hospitalization. Patient costs (calculated as the actual cost to treat the patient) included all supplies, labor, depreciation of equipment, etc., based on the sum of variable costs (direct) plus all fixed costs (overhead) as obtained from the premier alliance database.

## Results

A total of 351,601 patients (mean age 70.29 years) who experienced a stroke were identified for inclusion in this study, of whom 71,483 (20 %) had a secondary diagnosis of AF and 280,118 (80 %) had no record of AF. A summary of patient demographics, key clinical characteristics, and information relating to the health providers is reported in Table 1. Among patients hospitalized with ischemic stroke, AF was listed as a secondary diagnosis in 21 % (*n* = 59,172) and not listed as a secondary diagnosis in 79 % (*n* = 220,518). Among patients hospitalized with hemorrhagic stroke, 17 % (*n* = 12,311) and 83 % (*n* = 59,600) had and did not have a secondary diagnosis of AF, respectively. Among patients included in the study, there was a high prevalence of hypertension (63.5–68.2 %) and diabetes (21.2–29.6 %) in both AF and non-AF patient groups. However, while congestive heart failure (CHF) was present in ~31 % of patients with AF, only ~10 % of patients who did not have AF had this comorbidity.

**Table 1:** Demographic characteristics, comorbidities, and information relating to healthcare providers

	Stroke type, n (%)					
	Ischemic			Hemorrhagic		
	Non-AF (*n* = 220,518)	AF (*n* = 59,172)	*p* value*	Non-AF (*n* = 59,600)	AF (*n* = 12,311)	*p* value*
Age, years						
18–39	5,179 (2.4)	116 (0.2)	<0.0001	3,525 (5.9)	45 (0.4)	<0.0001
40–49	14,554 (6.6)	559 (0.9)		7,309 (12.3)	170 (1.4)	
50–59	34,523 (15.7)	2,393 (4.0)		11,801 (19.8)	653 (5.3)	
60–69	50,877 (23.1)	6,917 (11.7)		11,247 (18.9)	1,717 (14.0)	
70–79	56,588 (25.7)	15,987 (27.0)		11,824 (19.8)	3,738 (30.4)	
80+	58,797 (26.7)	33,200 (56.1)		13,894 (23.3)	5,988 (48.6)	
Sex						
Female	109,500 (49.7)	33,936 (57.4)	<0.0001	30,480 (51.1)	5,954 (48.4)	<0.0001
Race						
White	141,317 (64.1)	43,000 (72.7)	<0.0001	33,984 (57.0)	8,765 (71.2)	<0.0001
Black	33,594 (15.2)	4,515 (7.6)		10,047 (16.9)	847 (6.9)	
Hispanic	9,421 (4.3)	1,874 (3.2)		3,771 (6.3)	524 (4.3)	
Other	36,186 (16.4)	9,783 (16.5)		11,798 (19.8)	2,175 (17.7)	
Comorbidities						
Myocardial infarction	20,824 (9.4)	7,274 (12.3)	<0.0001	3,604 (6.1)	1,300 (10.6)	<0.0001
Congestive heart failure	23,146 (10.5)	19,052 (32.2)	<0.0001	4,729 (7.9)	3,558 (28.9)	<0.0001
Diabetes	65,191 (29.6)	15,793 (26.7)	<0.0001	12,655 (21.2)	3,353 (27.2)	<0.0001
Hypertension	15,0402 (68.2)	38,079 (64.4)	<0.0001	37,820 (63.5)	8,064 (65.5)	<0.0001
Provider characteristics						
Rural	21,781 (9.9)	5,722 (9.7)	0.1331	3,928 (6.6)	939 (7.6)	<0.0001
Urban	198,737 (90.1)	53,450 (90.3)		55,672 (93.4)	11,372 (92.4)	
Teaching hospital	88,392 (40.1)	23,752 (40.1)	0.8023	31,297 (52.5)	6,186 (50.3)	<0.0001
Beds						
0–99	5,892 (2.7)	1,688 (2.9)	<0.0001	559 (0.9)	172 (1.4)	<0.0001
100–199	23,697 (10.8)	6,515 (11.1)		4,015 (6.7)	930 (7.6)	
200–299	33,521 (15.2)	9,139 (15.4)		6,013 (10.1)	1,411 (11.5)	
300–399	50,575 (22.9)	13,423 (22.7)		11,568 (19.4)	2,484 (20.2)	
400–499	38,481 (17.5)	10,167 (17.2)		11,008 (18.5)	2,261 (18.4)	
500–599	25,217 (11.4)	6,718 (11.4)		7,777 (13.1)	1,683 (13.7)	
600–699	20,398 (9.3)	5,277 (8.9)		9,684 (16.3)	1,647 (13.4)	
700–799	10,048 (4.6)	2,446 (4.1)		3,216 (5.4)	572 (4.7)	
800–899	4,252 (1.9)	1,317 (2.2)		2,186 (3.7)	459 (3.7)	
900+	8,437 (3.8)	2,482 (4.2)		3,574 (6.0)	692 (5.6)	
Provider region						
Midwest	40,585 (18.4)	11,220 (19.0)	<0.0001	9,626 (16.2)	2,160 (17.6)	<0.0001
Northeast	40,941 (18.6)	12,313 (20.8)		13,039 (21.9)	2,993 (24.3)	
South	98,649 (44.7)	23,100 (39.0)		25,123 (42.2)	4,602 (37.4)	
West	40,343 (18.3)	12,539 (21.2)		11,812 (19.8)	2,556 (20.8)	

* Chi squared values for the variable, by presence of AF

*AF* atrial fibrillation

A summary of discharge status and mortality results is shown in Table 2. After adjusting for all covariates, patients with AF were more likely than patients without AF to be discharged to a continuing care facility following hospitalization for ischemic stroke (odds ratio [OR], 1.92; 95 % confidence interval [CI], 1.88–1.97; *p* < 0.0001) and hemorrhagic stroke (OR, 1.38; 95 % CI, 1.29–1.47; *p* < 0.0001). Patients with AF were also significantly more likely to die during the index hospitalization rather than be discharged home, irrespective of type of stroke (ischemic: OR, 3.49; 95 % CI, 3.35–3.63; *p* < 0.0001; hemorrhagic: OR, 1.58; 95 % CI, 1.47–1.69; *p* < 0.0001).

**Table 2:** Discharge status for patients with ischemic and hemorrhagic stroke in the premier alliance database

Effect	Comparison^a^	Discharged to continuing care			Died during hospitalization		
		Odds ratio	95 % Wald confidence limits	*p* value	Odds ratio	95 % Wald confidence limits	*p* value
Ischemic stroke							
AF	AF vs non-AF	1.92	1.88–1.97	<0.0001	3.49	3.35–3.63	<0.0001
Age, years	18–39 vs 80+	0.20	0.19–0.21	<0.0001	0.26	0.23–0.30	<0.0001
	40–49 vs 80+	0.22	0.21–0.23	<0.0001	0.22	0.20–0.25	<0.0001
	50–59 vs 80+	0.24	0.24–0.25	<0.0001	0.24	0.22–0.26	<0.0001
	60–69 vs 80+	0.27	0.26–0.28	<0.0001	0.24	0.22–0.25	<0.0001
	70–79 vs 80+	0.39	0.38–0.40	<0.0001	0.34	0.33–0.36	<0.0001
Sex	Male vs female	0.70	0.69–0.71	<0.0001	0.72	0.69–0.75	<0.0001
MI	Yes vs no	0.98	0.95–1.01	0.1255	1.63	1.55–1.71	<0.0001
CHF	Yes vs no	1.62	1.58–1.66	<0.0001	2.36	2.26–2.47	<0.0001
Diabetes	Yes vs no	1.28	1.25–1.30	<0.0001	1.19	1.14–1.24	<0.0001
Hypertension	Yes vs no	1.07	1.05–1.09	<0.0001	0.73	0.70–0.76	<0.0001
Race	Hispanic vs Black	0.70	0.67–0.74	<0.0001	0.85	0.76–0.94	0.002
	Other vs Black	0.59	0.57–0.61	<0.0001	0.70	0.65–0.75	<0.0001
	White vs Black	0.53	0.51–0.54	<0.0001	0.61	0.58–0.65	<0.0001
Hemorrhagic stroke							
AF	AF vs non-AF	1.38	1.29–1.47	<0.0001	1.58	1.47–1.69	<0.0001
Age, years	18–39 vs 80+	0.14	0.13–0.15	<0.0001	0.15	0.14–0.17	<0.0001
	40–49 vs 80+	0.19	0.17–0.20	<0.0001	0.21	0.19–0.23	<0.0001
	50–59 vs 80+	0.24	0.22–0.26	<0.0001	0.27	0.25–0.29	<0.0001
	60–69 vs 80+	0.32	0.30–0.35	<0.0001	0.34	0.31–0.36	<0.0001
	70–79 vs 80+	0.52	0.48–0.55	<0.0001	0.54	0.50–0.58	<0.0001
Sex	Male vs female	0.79	0.75–0.82	<0.0001	0.78	0.75–0.81	<0.0001
MI	Yes vs no	1.24	1.13–1.36	<0.0001	1.52	1.39–1.68	<0.0001
CHF	Yes vs no	1.61	1.49–1.75	<0.0001	1.48	1.36–1.61	<0.0001
Diabetes	Yes vs no	1.24	1.18–1.30	<0.0001	1.18	1.12–1.25	<0.0001
Hypertension	Yes vs no	1.37	1.31–1.43	<0.0001	1.15	1.09–1.21	<0.0001
Race	Hispanic vs Black	0.75	0.68–0.82	<0.0001	0.77	0.70–0.86	<0.0001
	Other vs Black	0.80	0.75–0.86	<0.0001	0.92	0.85–1.00	0.0449
	White vs Black	0.75	0.70–0.79	<0.0001	0.86	0.80–0.92	<0.0001

Odds ratios from multinomial regression. Data for hospital type and geographic region are not shown

*AF* atrial fibrillation, *CHF* congestive heart failure, *MI* myocardial infarction

^a^Patients classified as discharged to home were used as the reference group

Among patients with an ischemic stroke event, the duration of hospital stay was significantly longer for patients with AF (adjusted mean length of stay, 1.63 days longer; *p* < 0.0001). This was also true for patients who experienced hemorrhagic stroke (adjusted mean length of stay, 0.95 days longer; *p* < 0.0001). Table 3 shows a summary of the observed effect of various baseline characteristics and selected common cardiovascular comorbidities on the length of hospital stay.

**Table 3:** Length of stay for patients with ischemic and hemorrhagic stroke in the premier alliance database

Effect	Comparison	Difference (days)^a^	95 % CI	*p* value
Ischemic stroke				
AF	AF vs non-AF	1.626	1.614–1.637	<0.0001
Age, years	18–39 vs 80+	1.112	1.099–1.125	<0.0001
	40–49 vs 80+	0.536	0.532–0.540	<0.0001
	50–59 vs 80+	0.341	0.339–0.343	<0.0001
	60–69 vs 80+	−0.182	−0.181 to −0.183	<0.0001
	70–79 vs 80+	−0.259	−0.258 to −0.260	<0.0001
Sex	Male vs female	−0.115	−0.115 to −0.116	<0.0001
MI	Yes vs no	0.298	0.297–0.300	<0.0001
CHF	Yes vs no	1.265	1.260–1.271	<0.0001
Diabetes	Yes vs no	0.305	0.304–0.306	<0.0001
Hypertension	Yes vs no	−0.285	−0.284 to −0.287	<0.0001
Race	Hispanic vs Black	−0.175	−0.173 to −0.176	<0.0001
	Other vs Black	−0.712	−0.708 to −0.716	<0.0001
	White vs Black	−1.313	−1.307 to −1.320	<0.0001
Hemorrhagic stroke				
AF	AF vs non-AF	0.947	0.943–0.951	<0.0001
Age, years	18–39 vs 80+	4.149	4.099–4.201	<0.0001
	40–49 vs 80+	4.727	4.683–4.771	<0.0001
	50–59 vs 80+	4.135	4.101–4.169	<0.0001
	60–69 vs 80+	2.670	2.648–2.692	<0.0001
	70–79 vs 80+	1.530	1.518–1.542	<0.0001
Sex	Male vs female	0.273	0.272–0.275	<0.0001
MI	Yes vs no	0.130	0.129–0.132	0.0015
CHF	Yes vs no	2.253	2.236–2.270	<0.0001
Diabetes	Yes vs no	0.623	0.619–0.627	<0.0001
Hypertension	Yes vs no	0.533	0.530–0.536	<0.0001
Race	Hispanic vs Black	−0.661	−0.654 to −0.669	<0.0001
	Other vs Black	−0.863	−0.855 to −0.870	<0.0001
	White vs Black	−1.700	−1.688 to −1.712	<0.0001

Estimates from Poisson regression model. Data for hospital type and geographic region are not shown

*AF* atrial fibrillation, *CHF* congestive heart failure, *CI* confidence interval, *MI* myocardial infarction

^a^Absolute differences in length of hospital stay between AF and non-AF patients are given as the differences of least square means

Patients with AF had an adjusted mean patient cost associated with an ischemic stroke event that was $2,997.18 (95 % CI, $2,937.97–$3,057.58) higher than for patients without AF. For patients with AF having a hemorrhagic stroke event, this figure was $3,229.73 (95 % CI, $3,207.69–$3,251.91) higher than for those without AF. A summary of the effect of baseline characteristics and selected common cardiovascular comorbidities on patient costs associated with an index hospitalization for stroke is shown in Table 4.

**Table 4:** Patient costs for ischemic and hemorrhagic stroke in the premier alliance database

Effect	Comparison	Difference ($)^a^	95 % CI	*p* value
Ischemic stroke				
AF	AF vs non-AF	2,997.18	2,937.97–3,057.58	<0.0001
Age, years	18–39 vs 80+	4,542.61	4,452.69–4,634.34	<0.0001
	40–49 vs 80+	2,651.13	2,618.01–2,684.68	<0.0001
	50–59 vs 80+	2,009.55	1,991.55–2,027.71	<0.0001
	60–69 vs 80+	1,235.46	1,226.00–1,244.99	<0.0001
	70–79 vs 80+	745.56	740.34–750.82	<0.0001
Sex	Male vs female	308.80	307.14–310.47	<0.0001
MI	Yes vs no	1,228.62	1,217.79–1,239.56	<0.0001
CHF	Yes vs no	2,338.77	2,320.62–2,357.07	<0.0001
Diabetes	Yes vs no	473.68	470.87–476.50	<0.0001
Hypertension	Yes vs no	−536.92	−533.38 to −540.49	<0.0001
Race	Hispanic vs Black	1,188.85	1,170.98–1,206.99	<0.0001
	Other vs Black	−591.89	−585.92 to −597.93	<0.0001
	White vs Black	−1,087.18	−1,078.27 to −1,096.15	<0.0001
Hemorrhagic stroke				
AF	AF vs non-AF	3,229.73	3,207.69–3,251.91	<0.0001
Age, years	18–39 vs 80+	20,898.35	20,156.91–21,667.05	<0.0001
	40–49 vs 80+	20,204.50	19,671.12–20,752.35	<0.0001
	50–59 vs 80+	16,538.73	16,169.17–16,916.74	<0.0001
	60–69 vs 80+	11,945.59	11,687.14–12,209.76	<0.0001
	70–79 vs 80+	6,090.32	5,967.32–6,215.86	<0.0001
Sex	Male vs female	116.29	114.64–117.96	0.45
MI	Yes vs no	1,500.94	1,459.12–1,543.97	<0.0001
CHF	Yes vs no	6,485.87	6,336.96–6,638.27	<0.0001
Diabetes	Yes vs no	1,100.25	1,081.29–1,119.55	<0.0001
Hypertension	Yes vs no	198.47	195.18–201.82	0.269
Race	Hispanic vs Black	1,707.93	1,649.18–1,768.78	<0.0001
	Other vs Black	−1,136.94	−1,107.81 to −1,166.83	0.0001
	White vs Black	−2,494.88	−2,442.19 to −2,548.70	<0.0001

Estimates from the generalized linear model with gamma distribution and log link. Data for hospital type and geographic region are not shown

*AF* atrial fibrillation, *CHF* congestive heart failure, *CI* confidence interval, *MI* myocardial infarction

^a^The absolute differences in patient cost between AF and non-AF patients are given as the differences of least square means

Although the focus of this study was on the effects of AF, several other variables included in the analysis were associated with notable effects on patient outcomes. Among patients in the ischemic stroke group, hypertension was associated with a decreased likelihood of dying while in care versus being discharged home (OR, 0.73; *p* < 0.0001), as well as with a significantly decreased duration of stay (*p* < 0.0001) and patient costs (*p* < 0.0001). Conversely, in patients who experienced hemorrhagic strokes, hypertension was associated with an increased duration of stay (*p* < 0.0001) and an increased probability of dying while in care (OR, 1.15; *p* < 0.0001), although there was no significant effect on patient costs (*p* = 0.269). Patient costs and duration of hospital stay decreased with increasing patient age, which also correlated with a general trend toward an increasing OR of being discharged to care and of dying during the initial hospitalization. CHF was associated with an increased OR for dying while in care in both the ischemic stroke group (OR, 2.36, 95 % CI, 2.26–2.47; *p* < 0.0001) and the hemorrhagic stroke group (OR, 1.48; 95 % CI, 1.36–1.61; *p* < 0.0001).

## Discussion

This study analyzed a data source covering 4.5 years of hospital discharges, with over 350,000 strokes in 71,483 patients with AF and 280,118 patients without AF. Both ischemic and hemorrhagic stroke events in patients with AF were associated with a greater likelihood of being discharged to a continuing care facility, an increased risk of death from the stroke, a longer duration of hospital stay, and increased patient costs compared with stroke events in patients without AF.

Hypertension was associated with reduced duration of hospital stay and reduced costs in patients in the ischemic stroke group, but had the opposite effect on these outcomes in patients who experienced hemorrhagic stroke. This could be expected based on the pathophysiology of the different types of stroke. For example, an elevated blood pressure could be beneficial in helping to maintain blood supply during an ischemic event, but deleterious during a hemorrhagic or bleeding event. These opposite effects of hypertension might also help to confirm the validity of the data.

After controlling for other variables, increasing age was associated with a decrease in patient costs and duration of stay, irrespective of the type of stroke. However, it was also associated with an increased likelihood of dying while in care or of being discharged to further care rather than to home, both of which would result in the observed reduction in length of stay for the index hospitalization event. Additionally, as costs associated with additional care after the index hospitalization event were not included in this study, the shorter duration of the initial hospital stay for both outcomes could at least partly explain the apparent effect on patient costs.

Considering the aging population and that age is an important risk factor for stroke, the incidence of stroke is projected to increase over the next 20–30 years; this effect will be further magnified because age is a risk factor for AF [4, 5]. The synergistic combination of these two effects will likely lead to a greater incidence of stroke and a disproportionately greater incidence of AF-related strokes. Given the results of this study, increasing rates of AF-related stroke would increase the demand for nursing homes, skilled nursing facilities, and hospices, and raise the question of who will bear the financial burden of this trend. Under current Medicare guidelines, the cost of a skilled nursing facility is reimbursed as long as patients require follow-up care after a qualifying hospital stay, but only for up to 100 days [19]. If the stay extends beyond that, patients may use supplemental insurance coverage or would need to draw from personal resources until they meet Medicaid eligibility requirements. Thus, it is likely that an increase in AF-related strokes will impact a variety of payers, from the Federal Medicare and State Medicaid programs to private insurers and patients themselves.

Stroke risk is affected by nonmodifiable risk factors, such as age and sex, and modifiable risk factors, such as hypertension and high cholesterol levels [20]. Widespread use of low cost antihypertensives and statins, following a stricter implementation of current guidelines for these medications, could provide an excellent social return on investment with reduced rates of stroke and other cardiovascular events [21, 22]. AF is associated with an increased risk of stroke and more severe stroke outcomes than strokes unrelated to AF, and appropriate thromboprophylaxis with warfarin or one of the new oral anticoagulants (dabigatran, rivaroxaban, and apixaban) has been shown to significantly reduce stroke risk in patients with AF [23–26]. Therefore, a more rigorous implementation of current guidelines for anticoagulant use in AF could potentially offer substantial reductions in stroke events and associated costs in this patient population.

This analysis focused on stroke-associated outcomes in the presence or absence of AF, and no direct statistical comparisons were made between patients who experienced ischemic or hemorrhagic strokes. A key strength of this study was the large number of patients included in the database; however, there were also several inherent limitations regarding the level of detailed information available. First, whereas all patients included in the AF group had a history of AF prior to or on the date of their documented stroke, some cases of undiagnosed AF may have been included in the group that did not have AF. Second, characterization of comorbidities was limited to data captured during the hospitalization period; hence, the presence of pre-existing chronic conditions, such as valvular heart disease, diabetes, or hypertension would have been underestimated in the study population unless these conditions were specifically coded during the hospitalization event. Therefore, the impact of risk-modifying drug therapies used in an outpatient setting prior to hospitalization on the outcomes of interest could not be assessed, either. Third, the database was unclear as to whether the hospitalizations included primary admissions for stroke or whether patient transfers from other hospitals were included. Fourth, treatment differences were not taken into account and may have existed between groups, both before and after the stroke event. Fifth, the dataset did not include electronic medical record data on important clinical variables such as blood pressure, cholesterol levels or echocardiographic parameters, which could be explored in future research. Finally, information on duration of stay in continuing care facilities following hospital discharge or on any associated costs incurred during this time was unavailable.

Another potential limitation—that the study was retrospective and not prospective—could conceivably have introduced bias into the results. For example, CHF was seen in a higher proportion of patients with AF than without AF. This could be expected, as CHF is thought to predispose patients to AF, AF may predispose patients to CHF [27], and the prevalence of both is known to increase with age [28]. However, although the lack of a randomization step did result in some differences between categories, this was adjusted for as part of the multivariate model used in the analysis.

## Conclusions

Among patients hospitalized with either ischemic or hemorrhagic stroke, AF was associated with a higher mortality rate. Patient care was extended beyond the initial hospitalization in a greater proportion of patients with AF than without AF, and length of hospital stay and patient costs were significantly higher in patients with AF in both stroke subgroups. Further research is needed to determine how modifiable risk factors can be managed to improve outcomes in the US population.
